# Validation of the Chemotaxis of Plant Parasitic Nematodes Toward Host Root Exudates

**DOI:** 10.21307/jofnem-2019-063

**Published:** 2019-09-17

**Authors:** Wenshan Liu, Alexis L. Jones, Heather N. Gosse, Kathy S. Lawrence, Sang-Wook Park

**Affiliations:** Department of Entomology and Plant Pathology, Auburn University, Auburn, AL 36849

**Keywords:** cotton, *Heterodera glycines*, Host–parasitic relationship, Host specificity, *Meloidogyne incognita*, Motility assay, Peanut, *Rotylenchulus reniformis*, Soybean.

## Abstract

Plant parasitic nematodes (PPN) are microscopic soil herbivores that cause damage to many economic crops. For the last century, it has been proposed that chemotaxis is the primary means by which PPN locate host plant roots. The identities and modes of action of chemoattractants that deliver host-specific messages to PPN, however, are still elusive. In this study, a unique multidimensional agar-based motility assay was developed to assess the impacts of root exudates on the short-range motility and orientation of PPN. Three PPN (*Rotylenchulus reniformis*, *Meloidogyne incognita* and *Heterodera glycines*) and root exudates from their respective host and non-host plants (cotton, soybean, and peanut) were used to validate the assay. As predicted, *R. reniformis* and *M. incognita* were attracted to root exudates of cotton and soybean (hosts), but not to the exudates of peanut (non-host). Likewise, *H. glycines* was attracted to soybean (host) root exudates. These results underpinned the intrinsic roles of root exudates in conveying the host specificity of PPN. In particular, PPN selectively identified and targeted to hydrophilic, but not hydrophobic, fractions of root exudates, indicating that groundwater should be an effective matrix for chemotaxis associated with PPN and their host plant interactions.

Phytopathogenic nematodes are microscopic roundworms that develop obligate parasitic relationships with plants. Once sedentary endoparasitic nematodes reach a root surface, they insert their stomatostylet, enter root tissue, establish a feeding site near the vascular cylinder, and ingest cytosolic nutrients (Mitchum et al., 2013; Fosu-Nyarko and Jones, 2016). Damage caused by PPN is estimated to result in an annual loss of ~14% of world crop productions (Nicol et al., 2011), needing an urgent breakthrough in developing effective and sustainable pest management programs. It is however not necessarily forthcoming, due partly to a lack of our understanding of the modes of plant and PPN interactions. A current working model describes that PPN use chemotaxis to sense and locate host plant roots (Steiner, 1925; Prot, 1980; Van Dam and Bouwmeester, 2016), as they are motile animals undulating in the dorsal ventral direction (snake-like motion, Backholm et al., 2013). PPN develop longitudinal muscles and a thick cuticle as a hydrostatic skeleton, necessary for their locomotion, and are commonly thought to move through the soil a distance of ~1 m within their lifetime (Davis and MacGuidwin, 2005; Moore et al., 2010). However, it is still elusive whether the movement of PPN is autonomous or governed by environmental matrices such as water, insects, and/or animals, and is random or target specific toward chemoattractants associated with host plants.

In the current literature, various nematode motility assays have been conducted via employing agar gel (pluronic F-127), natural sand and soil as migration matrices, and elucidated that PPN are responsive to plant roots, pH, redox potentials, temperature, moisture, carbon dioxide, oxygen, and inorganic ions (reviewed in Prot, 1980; Perry, 1996; Curtis, 2008; Fosu-Nyarko and Jones, 2016). These studies, however, have failed to explain the host specificity of PPN, and argued that plant and PPN interactions are not selective in general (Prot, 1980). In contrast, it is widely accepted that PPN selectively target host, but not non-host, plant roots (Nicol et al., 2011). The most well characterized target selectivity of nematodes was described using maize roots and an entomopathogenic nematode, *Heterodera megidis* (Rasmann et al., 2005). In response to the feeding of western corn rootworm (WCR) larvae, maize roots emit a volatile compound (*E*)-β-caryophyllene to strongly attract *H. megidis* which in turn parasitizes and kills WCR larvae within a few days (Degen et al., 2004; Rasmann et al., 2005; Degenhardt et al., 2009). This indirect defense mechanism of maize sheds light on an intrinsic activity of root-derived allelochemicals (e.g., (*E*)-β-caryophyllene) in attracting selective nematodes. In line with this scenario, the soil supplement of charcoal hindered the invasion of host roots by PPN (e.g., *Meloidogyne incognita*, Peacock, 1961), together suggesting that discrete organic substances exuded from plant roots play important roles in conveying the host-specificity of PPN.

Recent studies have started to uncover the signaling and pharmacological activities of plant root exudates toward PPN (Venturi and Keel, 2016; Van Dam and Bouwmeester, 2016). For instance, potato cyst nematodes (e.g., *Globodera pallida* and *G. rostochiensis*) exhibited preferential relocations to potato root exudates over control solvents such as water or methanol. On the other hand, root exudates of pea and maize displayed stimulation of temporal paralysis to several phyto- and entomopathogenic nematodes such as *M. incognita*, *H. glycine*, *H. medigis*, *Steinernema feltia*, and *S. carpocapsae* (Devine and Jones, 2003; Farnier et al., 2012; Hiltpold et al., 2015; Jaffuel et al., 2015). Following recovery, the nematodes were then able to engage in pathogenicity, mobility, and environmental stability (Hiltpold et al., 2015).

Moreover, certain root exudates can impede the growth of, and further kill, PPN. Leafy vegetable crown daisy (*Bellis perennis*), when intercropped, exhibited a reduced infestation of *M. incognita* on tomato roots (Dong et al., 2014). Antimicrobial lauric acid, found in crown daisy root exudates, was proposed to be a nematicidal reagent, causing mortality of *M. incognita* at high concentrations (> 4 mM, Waters et al., 2003; Dong et al., 2014). The caveat is that lauric acid could also induce attraction of *M. incognita* at its lower, perhaps physiologically relevant, concentrations (< 2.92 mM, Dong et al., 2014). Further investigations will be needed to delineate an actual role and activity of lauric acid toward PPN. Nonetheless, these studies together suggest that, plants may utilize multiple and a combined activity of allelochemicals in orchestrating complex and concurrent communication nexus with numerous and different species of PPN, as well as other organisms (Devine and Jones, 2003; Farnier et al., 2012).

Indeed, root exudation is a predominant and active means to deliver plant messages to neighboring organisms and adjust rhizosphere reservoirs (Venturi and Keel, 2016). We thus hypothesized that PPN hijack the underground signaling network, and locate host plants by discrete semiochemicals produced and released by plant roots. To scrutinize this hypothesis, we assessed previously available nematode motility assays Clemens et al., 1994; Farnier et al., 2012; (Margie et al., 2013; Qi et al., 2015; Maleita et al., 2017) and further adjusted those bioassays to accommodate the direct contact (sense) and free-range movement of PPN toward or away from test compounds (e.g., root extracts and exudates). The specific objectives were to (i) develop a new and unique bioassay for PPN chemotaxis, and (ii) validate this bioassay using three PPN species (i.e., *R. reniformis*, *M. incognita*, and *H. glycines*) exposed to root extracts and exudates from host (cotton and soybean) and/or non-host (peanut) plants, which underpin the crucial properties of root exudates (esp., hydrophiles) in the host-specific recognition and orientation of PPN.

## Materials and methods

### PPN motility assay

**Fig. 1:**
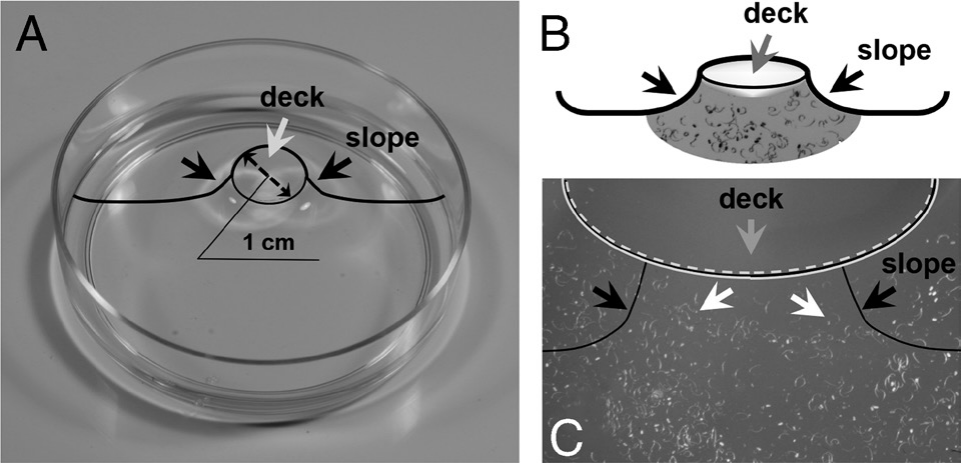
Figure 1: Development of a novel PPN motility assay. (A, B) Outline of an assay plate. A center of agar medium is uplifted to form a volcano-shaped, round deck (ø 1 cm). (B) Exemplary setup of a motility assay. PPN (e.g., *R. reniformis*, white arrows) suspended in water are placed around a slope of a volcano mountain, and carefully spread up to an edge of deck (white line), while a testing reagent (e.g., cotton root extracts, grey arrow) dissolved also in water is loaded on a volcano deck and spread to a top side of edge (white dash line). Subsequently, the reaction and movement of PPN are observed by a stereomicroscope and photographed. In (A to C), a shape of the volcano mountain is outlined by solid black line.

The bioassay was developed on the basis of an agar diffusion method, and conducted in an agar plate that forms a volcanic crater-like shape at its center (Fig. 1A). After pouring 0.2% (w/v) agar (plant cell culture tested, Sigma) in a small petri dish (50 mm diam.), the crater-shape was erected by capillary action, slightly lifting up a surface of agar medium (< 1 mm) using a paper or plastic straw (10 mm diam.). As outlined in Figure 1B, the center dome is referred to as the ‘volcano deck’ or ‘deck’, and the outer adjacent area skirt as the ‘volcano slope’ or ‘slope’. Note that the range of agar-medium concentrations used across the previous nematode motility assays (> 0.5%) appeared to be too hydrophobic to cause the aggregation of, and impede the movement of PPN. We therefore lowered agar concentrations down (to 0.2%) to increase the surface polarity, and to evade the surface tension of nematodes. However, further decreases in agar-medium concentrations (< 0.2%) debilitated medium solidity, and were disable to form the volcano deck. In order to maximize surface polarity of agar medium, agar plates were freshly prepared immediately before each assay.

Once the agar medium was polymerized and the assay plates located on a stereomicroscope (Olympus SZ40 or Nikon SMZ1500), ~300 freshly hatched PPN were pipetted in 20 μL H_2_O around the volcano slope (Fig. 1B and C). Subsequently, ~20 μL of the test compounds (root extracts, exudates, or water) were pipetted into the volcano deck. The movements of PPN were then recorded and photographed using microscope-mounted cameras (Cannon EOS Rebel T3i or Nikno DS-Fi1). During assays, the agar plates were covered with plate lids and black cloths to prevent water evaporation and potential light effect. Lastly, the number of PPN relocated onto a center of the volcano deck was recorded at 12 and 18 hr post co-incubation.

### Plant parasitic nematodes

*Rotylenchulus reniformis*, *M. incognita*, and *H. glycines* were cultured on the root of cotton, corn and soybean plants in the greenhouse. Forty five to 60 d after inoculation, cotton and corn roots were gently rinsed to remove the soil, and agitated for 4 min in 0.625% (w/v) NaOCl to extract the eggs of *R*. *reniformis* and *M. incognita* (Hussey and Barker, 1973). Soybean roots were washed over stainless-steel sieves (850 μm over 250 μm) to collect *H. glycines* cysts that were then grounded with a mortal and pestle to release eggs. The egg suspensions were passed through 75 μm over 25 μm sieves to remove debris. The eggs were hatched in a water-filled, modified Baermann funnel (Xiang et al., 2014) at 28˚C to 31˚C. Four to seven days after hatching, second-stage juveniles (J2) were collected on a 25 μm pore sieve, and enumerated at ×40 magnification using an inverted TS100 Nikon microscope and standardized to 30-40 J2 per 10 μL for the motility assays.

### Plant growth conditions

Cotton (germplasm LONREN-1, Robinson et al., 2007) soybean (Asgrow AG 5935) and peanut (Georgia 09B, FloRun107 and TifGuard) were grown in a chamber with a 12-hr day cycle at 25 ± 2˚C and 60% to 80% relative humidity.

### Root extract preparation

Roots of 3-wk-old cotton and peanuts were water-rinsed to remove the soil, immersed in liquid N_2_ and ground to a powder using a mortar and a pestle. Ground root tissues were dissolved into three volumes of 5% (v/v) MeOH, thoroughly homogenized, and centrifuged for 10 min at 10,000*g*. The supernatant (referred as ‘root extracts’ hereafter) was pelletized using the Speed-Vac, and finally suspended in H_2_O. The total root extracts were stored at 4°C until use.

### Root exudates preparation

After harvesting 2 to 3-wk-grown plants, soil was carefully dislodged from the roots under tap water, and plants were placed in a 1 L beaker containing H_2_O for 24 hr to collect exudates. The beaker mouths were taped across four times using a labeling tape where the plant leaves were located, so root but not stem and leaf tissues were submerged into the water. The root exudates were subsequently filtered by several layers of cheesecloth and cellulose filter paper (CFP4) to remove the root debris and soils, then freeze-dried and stored at -80^o^C until use.

For use in the PPN motility assay, the powders of root exudates were resuspended into H_2_O (1 mL), or further separated into hydrophilic (polar) supernatants and hydrophobic residues by being suspended in 5% (v/v) MeOH (1 mL) and centrifuged at 8,500 rpm for 15 min. Hydrophilic supernatants were then desiccated in a Speed-Vac with heat (~40^o^C), and both hydrophilic and hydrophobic residues were resuspended in H_2_O (0.5 mL) before use.

### Validation of PPN motility assays

Initial validation of the PPN motility assay was conducted with *R. reniformis* and cotton (LONREN-1) root extracts, and subsequently between *R. reniformis* and root extracts prepared from three peanut varieties (Georgia 09B, FloRun107 and TifGuard). The next experiments were then carried out with *R. reniformis* and the root exudates of cotton plants (LONREN-1). The cotton root exudates were tested by total exudates, and hydrophilic and hydrophobic fractions separated in 5% (v/v) MeOH. The final validation of the PPN motility assay was performed using three PPN species (i.e., *R. reniformis, M. incognita* and *H. glycines*) and root exudates from their hosts (cotton, LONREN-1, and soybean, Asgrow AG 5935) and/or non-host (peanut, Georgia 09B) plants. Water was included as a negative control.

**Fig. 2:**
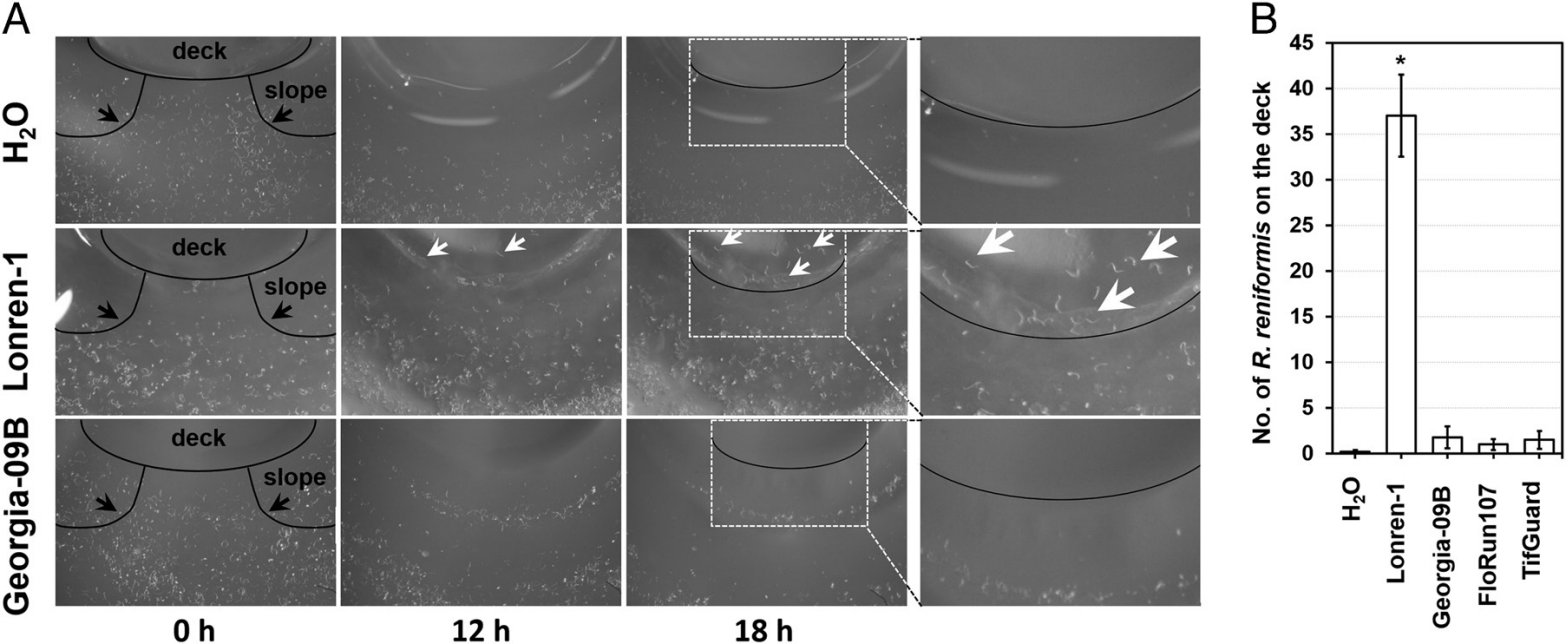
Figure 2: Validation of a novel PPN motility assay; *R. reniformis* is attracted toward cotton root extracts, but does not respond to peanut root extracts. (A) The time-resolved responses of *R. reniformis* upon the exposure to water, and root extracts prepared from 2-wk grown cotton plants (LONREN-1) and 3-wk old commercial peanut variety (Georgia-09B). Representative photographs are taken at 0, 12 and 18 hr of assays. Close up pictures of the boxed sections in 18 hr were shown in the right panel. Black lines draw the shapes of the volcano mountain, and white arrows indicate *R. reniformis* on the volcano deck. (B) Chemotactic behaviors of *R. reniformis* toward the root exudates of cotton and peanut plants. Numbers of *R. reniformis* relocated onto the volcano deck were counted at 18 hr post co-incubation with water and root extracts prepared from 2-wk grown cotton plants (LONREN-1) and 3-wk old commercial peanut varieties (Georgia-09B, FloRun107 and Tif Guard) (one-way ANOVA, *N* = 5). Asterisks (*) indicate statistically significant differences of chemotactic behaviors of *R. reniformis* toward the selected root extracts in comparison to water control by Dunnett’s *P* < 0.05.

All data were subjected to statistical analyses of variance using the SigmaPlot software. The significant levels of data presented in Figure 2B and Table 1 were compared by One-way ANOVA (*N* = 5 or 4, *P* < 0.05).

**Table 1.** Chemotactic behaviors of PPN toward the root exudates of host and nonhost plants.

Root exudates	Numbers of PPN relocated onto the volcano deck			
PPN	Cotton	Soybean	Peanut	Water
*R. reniformis*	13.5 ± 6.9*	10.0 ± 5.6*	0.5 ± 0.6	0
*M. incognita*	21.7 ± 6.2*	17.3 ± 3.5*	0	1.0 ± 1.4
*H. glycines*	3.0 ± 3.8	26.6 ± 9.8*	12.3 ± 6.2	0

Note: The significant levels of all data were compared by one-way ANOVA (N ; 4) in the SigmaPlot. Asterisks (*) indicate statistically significant differences of chemotactic behaviors of each PPN toward the selected root exudates in comparison to water control by Dunnett's *P* < 0.05

### Number of replicates

**Fig. 3:**
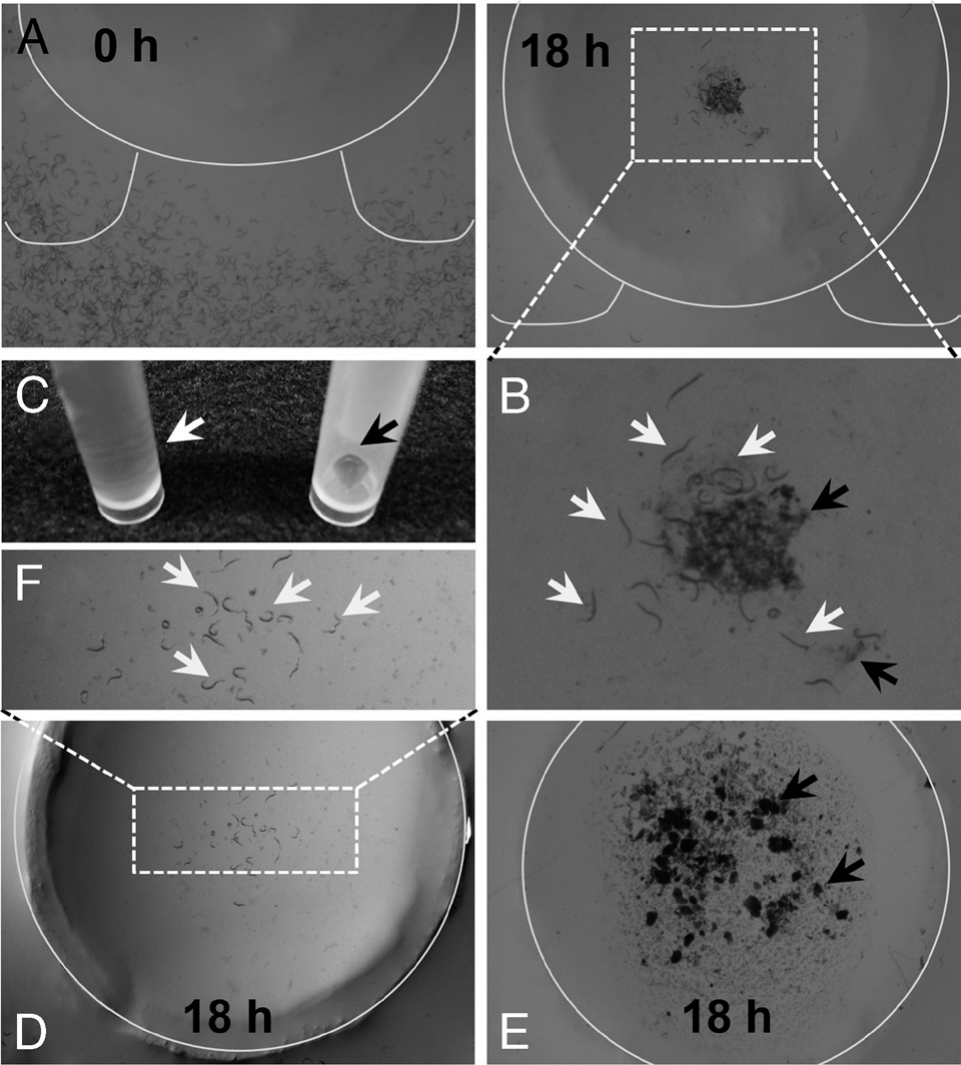
Figure 3: Root exudates of cotton plants attract *R. reniformis*. (A) Relocation of *R. reniformis* towards cotton root exudates. The time-resolved movement of *R. reniformis* toward cotton (LONREN-1) root exudates. Representative photographs are taken at 0 and 18 hr of assays. (B) Close up picture of a boxed section in (A at 18hr). White arrows indicate *R. reniformis*, and black arrows indicate water-insoluble precipitates of cotton root exudates on the deck. (C) Separation of hydrophilic supernatant (white arrow) and hydrophobic residue (black arrow) from total cotton root exudates (D, E). Responsive behaviors of *R. reniformis* toward hydrophilic (polar, D), and hydrophobic (nonpolar, E) compounds of cotton root exudates at 18 hr of assays. Polar, but not nonpolar, compounds of cotton exudates were able to attract *R. reniformis*. (F) Close up picture of a boxed area in (D), and white arrows indicate *R. reniformis* on the deck. In (E), black arrows indicate dark residues, partially water-undissolved nonpolar compounds of root exudates, which did not attract *R. reniformis*. In (A, D and E), white lines draw the shapes of the volcano mountain.

The experiments shown in Figures 2A, 2B, 3A, and 3B were repeated at least four times with similar results, while those shown in Figures 3C to 3F were performed three times with similar results. Each biological replicate was conducted with at least five agar-plates (*N* ≥ 5). Table 1 summarizes five (*R. reniformis*) and three (*M. incognita* and *H. glycines*) biological replicates (*N* ≥ 5). Note that each biological replicate used nematodes isolated and hatched independently.

## Results

### *R. reniformis* locates cotton, but not peanut, root extracts

To validate and optimize the PPN motility assay, we first tested it in assessing the responsive behaviors of *R. reniformis* toward root extracts prepared from its host (cotton) or non-host (peanut) plants (Fig. 2A and B). In the control assays with water, *R*. *reniformis* gradually dispersed away from the volcano deck with no *R. reniformis* on the volcano deck after 18 hr (Fig. 2A). Since water is chemostatic, the relocation of *R*. *reniformis* occurred likely by gravity on a slope of the volcano deck, rather than the repellent or toxic activity of water. In comparison, *R. reniformis* (~12% of the population) steadily traveled onto the volcano deck when the extracts of cotton roots were used, but exhibited little if any attraction to the volcanic deck containing the root extracts of peanuts (non-host plants). Most *R. reniformis* (> 99%) moved down and migrated away from the volcano deck when root extracts of three peanut varieties were examined (Fig. 2A and B). Together, these results elucidated that PPN are chemotactic toward metabolites produced in host plant roots, and can move actively and autonomously toward chemoattractants.

### Cotton root exudates attract *R. reniformis*

To further substantiate the physiological relevance of the initial results, we investigated if the root-derived chemoattractants of PPN could be released as the parts of root exudates so that they are available to rhizosphere for contacting PPN. Toward that, cotton root exudates were prepared as described in Materials and Methods and subjected to the motility assays along with *R. reniformis* (Fig. 3A and B). After 18 hr of co-incubation, a group of *R. reniformis* ascended the volcanic slope and congregated on the center of the volcano deck, indicating that plant roots produce and are able to exude the discrete chemoattractant(s) of PPN. In particular, the polar (hydrophilic) compounds in root exudates conveyed a key activity in signaling *R. reniformis* (Fig. 3C to F). During our assays (e.g., Fig. 3A and B), we started to notice that cotton root exudates resuspended in water slowly yielded precipitates (nonpolar compounds, see black arrows in Fig. 3B), and initially speculated that those precipitates include ‘active’ chemoattractant(s) because organic substances are largely water-insoluble. However, subsequent motility assays testing singly hydrophilic or hydrophobic fractions of cotton root exudates (Fig. 3C) displayed that the polar, but not nonpolar, fraction of cotton root exudates was able to attract *R. reniformis* (Fig. 3D to F).

### PPN are able to discern and target the root exudates of own host plants

Our results demonstrated an intrinsic activity of root-exuded allelochemicals in plant and PPN interactions. Hence, we attempted to access whether root exudates convey a host specificity of PPN, by cross-examining the responsive behaviors of three most destructive PPN (i.e., *R. reniformis*, *M. incognita*, and *H. glycines*) toward the polar factions of root exudates prepared from their host and non-host crops (cotton, soybean and peanut) (Table 1). As expected, *R. reniformis* and *M. incognita* were attracted to root exudates of cottons and soybeans (host plants), but not peanuts (non-host). In parallel, *H. glycines* migrated mainly toward soybean (host plant) root exudates. These observations concurred with a conclusion that PPN can discern host plants through sensing selective chemoattractants in root exudates, in particular polar compounds such as organic and amino acids, and peptidic and nucleotide-containing metabolites (Bertin et al., 2003; Pétriacq et al., 2017).

## Discussion

A century ago, Steiner (1925) proposed that PPN locate their host plants through chemoreception. Subsequently, a series of bioassays showed that plant-derived allelochemicals could directly or indirectly (via modifying rhizospheric states), positively or negatively control short-distance communications between plant roots and PPN (Prot, 1980; Curtis, 2008; Van Dam and Bouwmeester, 2016). It is still elusive how PPN discern host plants, as most published studies have described the chemotaxis of PPN toward host-nonspecific stimuli such as rhizospheric gradients in temperature, moisture, pH or redox potentials, and plant-derived mineral salts, carbon dioxide, or oxygen (Prot, 1980; Perry, 1996). Thus, earlier reports once proposed that an orientation of PPN is not decided by particular attractants (Prot, 1980; Wieser, 1956), but rather determined via the ratios of attractants and repellents exuded from plant roots (Wieser, 1956; Castro et al., 1989; Diez and Dusenbery, 1989; Devin and Jones, 2003). It was an intriguing idea to illustrate how PPN can pick and attack selective target plants. This hypothesis however was still unable to fully explain a host specificity of PPN, because there is little or no evidence that those repellents are PPN- and/or host-specific. Hence, plants secreting higher attractants ratios to repellents could render susceptibility, whereas secreting lower or equal attractants ratios to repellents likely confer resistance to most of all, but not selective, PPN.

Alternatively, recent studies have proposed so-called ‘searching behavior’ of PPN (e.g., *Heterodera* spp. and *Meloidogyne* spp.). When tested with plant roots and exudates, PPN displayed an ability to not only detect but also discern the gradients of chemical cues, which enables them to preferentially orient and take shorter (or more effective) routes to reach the source of chemoattractants, host roots, and exudates (Papademetrious and Bone, 1983; Clemens et al., 1994; Reynolds et al., 2011; Farnier et al., 2012; Hu et al., 2017). In fact, PPN are able to identify distinct allelochemicals in root exudates. When the reactions of potato cyst nematodes (PCN; e.g., *G. rostochiensis*) were assessed in regards to fractions of potato root exudates separated by an ion-exchanger chromatography, PCN responded only toward a subset of the fractions (6/30, Devine and Jones, 2003). This suggests the presence of discrete allelochemical attractants in the root exudates, intrinsic in the plant root and PPN interactions. These chemoattractants are then disseminated via soil as aqueous solutions and possibly, rhizospheric pores as gaseous compounds to excite the sensory mechanisms of PPN that cue chemotactic responses (Rolfe et al., 2000; Farnier et al., 2012). Indeed, allelochemicals released as part of root exudates can travel through soils up to ~10 cm from the sources (Hiltpold and Turlings, 2008).

In line of this scenario, our new motility assays demonstrate that the root-derived organic substances convey a host specificity of PPN, as they differentially locate the root exudates of host plants vs. non-host plants (Table 1). For instance, southern nematodes, *R. reniformis* and *M. incognita*, responded and moved only toward the root exudates of cotton and soybean, but not peanut plants. The results led us to speculate that those PPN from distant genera still could share the same or similar host ranges through perceiving the same chemoattractants (signal compounds) released by host, but not non-host, plant roots. On the other hand, *H. glycines* displayed attraction mainly toward soybean, indicating that its chemotaxis is stimulated by different signal compounds from those for *R. reniformis* and *M. incognita*, or by different level sensitivity toward similar or same signal signatures across major host vs. other plant roots. Nonetheless, these signal compounds are mainly hydrophiles (Fig. 3). Hydrophiles can be dissolved and distributed via soil water across rhizospheres. Moreover, boiling caused little if any effect on the attractant activities of roots exudates (data not shown), further suggesting that the signal compounds are perhaps nonpeptidic, organic acid metabolites. Together, the results highlighted the presence and key roles of chemoattractants in plant root exudates, which orchestrates the host-specific communications between plants and PPN.

It is worth noting that other studies have hypothesized that PPN ‘learn and memorize’ semiochemical cues at hatching and search for these cues once becoming of infective ages (e.g., J2 juvenile; Clarke and Hennessy, 1984; Rankin et al., 1993; Clemens et al., 1994; Devine and Jones, 2003). However, a number of bio- and pathoassays using neutral solvents (e.g., water) to hatch eggs and grow juveniles have not observed (or reported) significant loss of the host-specificity and infestation capacity of PPN (e.g., Sikkens et al., 2011; Xiang and Lawrence, 2016). This indicates that a prior orientation is not essential for the chemotaxis (host-specificity) of PPN. Alternatively, PPN may carry chemoreceptors to discern external signals, and root exudates (Rolfe et al., 2000; Wicher, 2012). In the J2 stages, a cyst nematode, *H. glycines* accumulates a guanylyl cyclase-2 (GC-2; a homolog of *Caenorhabditis elegans* chemoreceptor) around the sensory neurons of its amphids and caudal region (Yan and Davis, 2002; Bergmann, 2006). This may describe the searching behavior, toward host-nonspecific stimuli such as inorganic ions, carbon dioxide, and thermosensation (Papademetrious and Bone, 1983; Maruyama, 2017). Indeed, those carbon dioxide and nutritious substances can also serve as a beacon of rhizophagous insect herbivories through stimulating receptive sensilla located in the palpal apices of their mouthparts (Doane and Klingler, 1978; Steeghs et al., 2004; Reinecke et al., 2008; Eilers et al., 2012). However, little is known about the PPN chemoreceptors; further investigations are needed to understand the roles and activities of GCs, and other chemoreceptor candidates such as G-protein coupled receptors, nuclear receptors, and kinases (Abad et al., 2008; Opperman et al., 2008; Schaff et al., 2011) in the modes of host-specific recognition by PPN, and the subsequent transmission of sensory signals that control the responses and movements of PPN.

As an initial step to further delineate the chemotaxis of PPN, this study developed a unique agar-based, multidimensional motility assay, and validated that PPN can intercept the signaling nexus of plant rhizospheres and locate host plants by distinct chemoattractants (esp. hydrophiles) released via root exudates. The bioassay enabled nematodes to adequately sense and react to testing reagents (e.g., root extracts and exudates). A crucial drawback of previous bioassays was their failure to accommodate the direct/close interactions between PPN and test chemicals, because most, if not all, bioassays require suitable inorganic solvents (e.g., water) to resuspend and handle both PPN and testing compounds. When two solvents were applied adjacent to each other, they became cohered and blended; disabling to monitor the responsive behaviors of PPN toward testing compounds. Conversely, the new bioassay allowed two solvents to adhere to/throughout the rim of volcano deck and slope (Fig. 1C, white dash or straight line), without causing the solvent cohesion. Hence, PPN were positioned in close proximity to testing reagents could perceive them through agar-media pores in the rim of volcano deck and slope, offering a new methodology to investigate the semiochemical activity of involatile compounds toward PPN. In addition, the ascending of PPN onto the volcano deck, against gravity, in search of chemoattractants (e.g., root exudates) must cost the PPN more energy than that used in random migration of an equivalent distance on a flat surface. This underpins the specific interactions between e.g., *R. reniformis* and cotton root exudates, (Fig. 3), and the utility of our motility assay in discerning and/or searching the ‘true’ chemoattractants of PPN.
